# Nucleic acid testing in Colombian blood banks (2018–2024): Implementation, yield estimates and implications for safer transfusion policy

**DOI:** 10.1111/vox.70078

**Published:** 2025-08-06

**Authors:** María‐Isabel Bermúdez‐Forero, Michel‐Andrés García‐Otálora

**Affiliations:** ^1^ Instituto Nacional de Salud (INS) Coordinación Red Nacional Bancos de Sangre y Servicios de Transfusión Bogotá Colombia; ^2^ School of Medicine and Health Science, Public Health Research Group Universidad del Rosario Bogotá Colombia

**Keywords:** haemovigilance, HBV, HCV, HIV, NAT

## Abstract

**Background and Objectives:**

Colombia has a population of 52.6 million inhabitants and a blood donation rate of 26 donations per 1000 inhabitants. The 84 blood banks in the country collect approximately 1 million donations annually, which are mandatorily screened for human immunodeficiency virus (HIV), hepatitis B virus (HBV), hepatitis C virus (HCV), human T‐lymphotropic virus (HTLV), *Trypanosoma cruzi* and *Treponema pallidum*. Currently, 95% of donations are screened using chemiluminescence and the remaining using enzyme‐linked immunosorbent assays (ELISA). However, nucleic acid testing (NAT) is not mandatory for any infectious agent. The aim was to assess the progress in the voluntary implementation of NAT for HIV, HBV and HCV in blood banks.

**Materials and Methods:**

Data from the National Haemovigilance Information System were analysed between January 2018 and December 2024, including the total number of donations collected and the screening results obtained through chemiluminescence, ELISA as well as NAT in blood banks that voluntarily adopted this technology.

**Results:**

During the study period, a total of 6,435,189 blood units were collected, of which 6.9% were screened using NAT. The data revealed 10 undetected immunological windows with conventional techniques: four for HIV, three for HBV and three for HCV. It is estimated that the universal implementation of NAT would have identified 135 additional infectious units, potentially preventing at least 83 infections in recipients.

**Conclusion:**

The progressive implementation of NAT in Colombian blood banks has demonstrated the presence of immunological windows undetectable by conventional serological techniques, highlighting the potential risk for blood recipients. These findings underscore the need to accelerate NAT adoption and consider making it mandatory in 100% of the country's blood banks.

**Highlights**

- In Colombia, from 2018 to 2024, 6,435,189 blood units were collected, but only 6.9% were screened using nucleic acid testing (NAT). NAT detected 10 cases (four human immunodeficiency virus, three hepatitis B virus and three hepatitis C virus) that were missed by chemiluminescent immunoassay or enzyme‐linked immunosorbent assay, equating to 2.3 NAT‐yields per 100,000 donations.
- Modelling based on NAT‐yield rates suggests that, under serology‐only screening, a theoretical residual risk of 8.6 per million components transfused remains.
- Surveillance data revealed 169 confirmed transfusion‐related infections (observed residual risk of 17.54 per million components), while only 10 were reported through the official haemovigilance system, underscoring both the limitations of serological screening and gaps in national reporting.

## INTRODUCTION

In Colombia, 84 blood banks collect nearly 1 million blood units each year (national donation rate: 26 per 1000 inhabitants) [1]. Blood donation is regulated by Decree 1571 (1993) [2], which mandates that all blood units must undergo serological screening for human immunodeficiency virus (HIV)‐1/2 (antigen and antibody assays), hepatitis B surface antigen (HBsAg), antibodies against hepatitis C virus (HCV) and *Treponema pallidum*. Subsequent resolutions—1738 (1995) [3] and 437 (2014) [4]—expanded these requirements to include tests for *Trypanosoma cruzi*, hepatitis B core antigen (Anti‐HBc), and human T‐cell lymphotropic virus (HTLV). By the end of the first half of 2024 [5], most blood banks (95.1%) used chemiluminescent or electrochemiluminescent immunoassays (CLIA/ECLIA) for these screenings, while 4.9% still relied on fourth‐generation enzyme‐linked immunosorbent assays (ELISA).

Nucleic acid testing (NAT) is a molecular technique that enhances blood safety by significantly reducing the time to detection of the targeted pathogen, thereby shortening the window period during which infections may go undetected by serological methods. Introduced in Germany in 1997 for the detection of HIV, hepatitis B virus (HBV) and HCV, NAT has since become integral to global blood donation screening [6, 7]. Despite its proven efficacy—evidenced by the detection of numerous infections missed by serological tests—the high cost of NAT has limited its universal adoption in some regions [7].

In Latin America, Brazil is the only country that mandates NAT for all blood donations [8, 9]. As of early 2025, Colombia has not implemented universal NAT screening. A 2016 systematic review by the Ministry of Health assessed whether NAT could effectively reduce the risk of transfusion‐trasmitted infections (TTIs) caused by HBV, HCV and HIV. The review found that NAT outperforms immunoassay tests by shortening the window period of these viruses [10]. Building on this evidence, a 2018 cost–utility study recommended integrating NAT, emphasizing not only its clinical advantages but also its ethical and social benefits in protecting recipients' health [11].

Between 2018 and 2023, Colombia experienced increases in both the incidence and prevalence of HIV (43.2% and 72.8%) [12], HBV (16.5% and 42.9%) [13, 14, 15] and HCV (156.8% and 130.1%) [16], particularly among males aged 15–64 years. Additionally, blood bank data reveal persistent reactivity rates for HIV, HBsAg and HCV antibodies (in 2023: 0.2%, 0.1% and 0.3%, respectively) [1]. Notably, the National Haemovigilance System (Sistema Nacional de Hemovigilancia del Instituto Nacional de Salud [SIHEVI‐INS]) recorded 10 cases of transfusion‐transmitted HIV infections during this period [17, 18, 19], while no cases of transfusion‐transmitted HBV or HCV have been reported.

In addition, since 2021, the permanent deferral of certain populations as blood donors has been lifted [20]. In response, the National Network of Blood Banks, at the Colombian National Institute of Health (INS)—which is responsible for coordinating all blood banks—issued a new regulation on NAT in 2023 [21]. This regulation mandates that serological screening using CLIA/ECLIA/ELISA must continue at 100%, with blood banks performing NAT on‐site required to maintain parallel serological screening, follow manufacturer protocols, validate procedures and apply defined algorithms for donors with positive results. Blood banks that do not have the infrastructure to implement NAT on‐site are permitted to outsource it to other accredited blood banks. To ensure timely results, the referring bank may send prior immunoassay screening results to the processing bank to facilitate the formation of NAT pools. However, several responsibilities must be undertaken: first, the referring bank retains full responsibility for the quality and safety of the blood components, including traceability and compliance with national standards; second, both institutions must establish and document protocols for sample packaging, transportation, custody, quality control monitoring and the secure and timely transmission of results; third, the receiving bank, which performs NAT, is responsible for maintaining a sample archive of processed donations under conditions recommended by the manufacturer to ensure proper storage in case haemovigilance investigations are needed; and lastly, pool formation must adhere strictly to the instructions provided by the test manufacturer, prioritising process safety and blood release protocols.

This study aimed to describe the implementation and coverage of NAT in Colombia between 2018 and 2024, estimate NAT‐yield rates and assess the potential impact of expanding NAT screening on transfusion safety.

## MATERIALS AND METHODS

### Study design and data sources

This retrospective observational study was conducted using data collected in SIHEVI‐INS from 84 blood banks and 650 clinics across Colombia between January 2018 and December 2024. Data extraction was performed using standardized forms, and all records were anonymized prior to analysis to ensure confidentiality.

### Serological screening procedures

**FIGURE 1:**
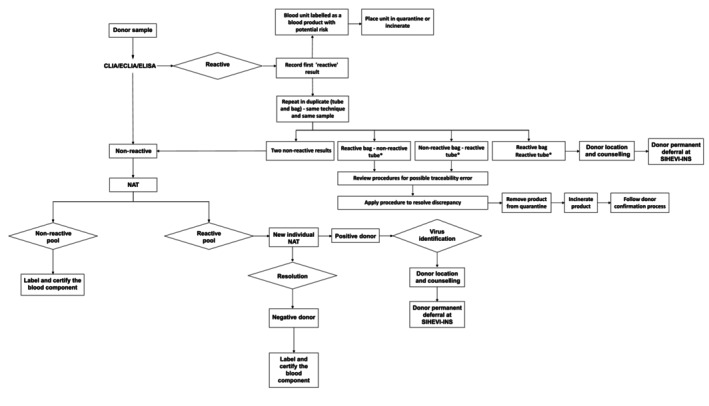
Algorithm for blood donor screening using nucleic acid testing (NAT) and serological methods in Colombia. This flowchart illustrates the decision‐making process for screening blood donations. All donor samples undergo both serological testing (chemiluminescent immunoassays/electrochemiluminescent immunoassays/enzyme‐linked immunosorbent assays [CLIA/ECLIA/ELISA]) and NAT. Serological reactive results are confirmed by duplicate testing. If both repeats are non‐reactive, the unit is cleared; otherwise, it is quarantined or discarded. NAT is performed using six‐sample minipools—non‐reactive pools lead to component release, while reactive pools undergo individual testing. Confirmed NAT‐reactive and serology‐negative cases are classified as NAT‐yield, prompting virus identification, donor counselling and permanent deferral in Sistema Nacional de Hemovigilancia del Instituto Nacional de Salud (SIHEVI‐INS). Discrepancies between bag and tube results are investigated for traceability errors and resolved through predefined protocols. *NAT is also performed on these samples; however, regardless of whether the result is reactive or non‐reactive, the unit is discarded, the donor is located and provided with counselling, and the donor is deferred in the SIHEVI‐INS system.

Quality control measures included cross‐checking data entries and validating laboratory results against established national protocols. For serological testing, the INS requires repeating tests to reduce false positives. Specifically, if a serological screening test initially yields a reactive result, it must be retested in duplicate. If both repeat tests are non‐reactive, the donor sample is considered negative. However, if one or both repeat tests remain reactive (classified as repeatedly reactive), the blood products cannot be used for allogeneic transfusion and must be discarded, the donor is deferred in SIHEVI‐INS [22] and confirmatory tests are conducted (Figure 1).

### Nucleic acid testing implementation

In blood banks where NAT for HIV, HBV and HCV was implemented, parallel serological testing using CLIA/ECLIA or ELISA assays was performed according to the manufacturers' protocols (Figure 1). This process used polymerase chain reaction (PCR) with a six‐plasma EDTA samples pooling strategy. Nationwide, all blood banks employed the cobas Multiplex (MPX) system [23], along with control kits and the Hamilton Microlab Star pipetting system. NAT assays were conducted in compliance with quality control standards. For the purposes of this paper, we define a NAT‐yield as a donation that tests reactive in both the initial pool and subsequent individual donation NAT but non‐reactive by serology, as published by Stramer et al. [24] and Faddy et al. [7]. The 95% limits of detection in EDTA plasma for individual samples were 25.7 IU/mL for HIV‐1 Group M, 8.2 IU/mL for HIV‐1 Group O, 4.0 IU/mL for HIV‐2, 1.4 IU/mL for HBV and 7.0 IU/mL for HCV. In six‐sample minipools, these limits were 154.2, 49.2, 24.0, 8.4 and 42 IU/mL, respectively.

### Pooling strategy and sensitivity considerations

To balance cost, throughput and detection performance, blood banks adopted a six‐sample minipool approach on the cobas MPX platform. Pooling reduces reagent use and processing time but increases the assay's limit of detection roughly six‐fold compared to individual testing (e.g., from 7 to 42 IU/mL for HCV). This elevated limit may slightly extend the serological window period and risk missing very low‐level viraemia. However, Galel et al. demonstrated that the cobas MPX assay has high specificity when testing both whole blood and source plasma donations, whether performed individually or in six‐sample pools [23]. All donation samples within a reactive minipool are tested individually to identify both the sample that was reactive and the viral cause of the reaction [24]. By cross‐referencing minipool results with individual limits of detection, we estimate that the sensitivity loss remains within acceptable bounds for public health surveillance while enabling broader NAT coverage at reduced cost.

### Donor‐type subanalysis

To assess whether NAT‐only reactivity differed between first‐time and repeat blood donors, we conducted a stratified analysis of all donations screened with NAT. Donations were classified according to donor type as either first‐time or repeat, and NAT‐yield was defined as donations that tested reactive by NAT but non‐reactive by CLIA/ECLIA/ELISA. NAT‐yield rates per 100,000 donations were calculated separately for each group. We compared proportions using the chi‐square test and Fisher's exact test (two‐sided), as appropriate. Risk ratios (RRs) were calculated to estimate the strength of association. Additionally, a logistic regression model was fitted to estimate the odds ratio and 95% confidence interval (CI) for repeat versus first‐time donor status, using NAT‐only reactivity as the dependent variable.

### Residual risk estimation

To estimate the number of transfusion recipients potentially exposed to HIV, HBV or HCV infections despite routine serological screening, we applied two complementary approaches.

First, we used a theoretical extrapolation model based on the NAT‐yield. We first determined the number of donations that were specifically screened using NAT and, among these, identified the number of NAT‐yield cases. We then calculated the NAT‐yield rate per 100,000 donations. Subsequently, we subtracted the NAT‐screened donations from the total number of donations collected during the study period and projected the number of potentially infectious units that might have gone undetected under a serology‐only screening strategy. Using the average number of components derived from whole‐blood donations and the average number of components transfused per patient, we estimated the number of potentially exposed patients.

Second, we used surveillance data from the Colombian High‐Cost Account (Cuenta de Alto Costo), which reported confirmed cases of HIV [12, 25, 26] and HCV [16, 27] attributed to transfusion during the same period. We calculated the residual risk of TTIs by dividing the total number of confirmed cases by the number of blood components transfused from 2018 to 2024.

## RESULTS

**FIGURE 2:**
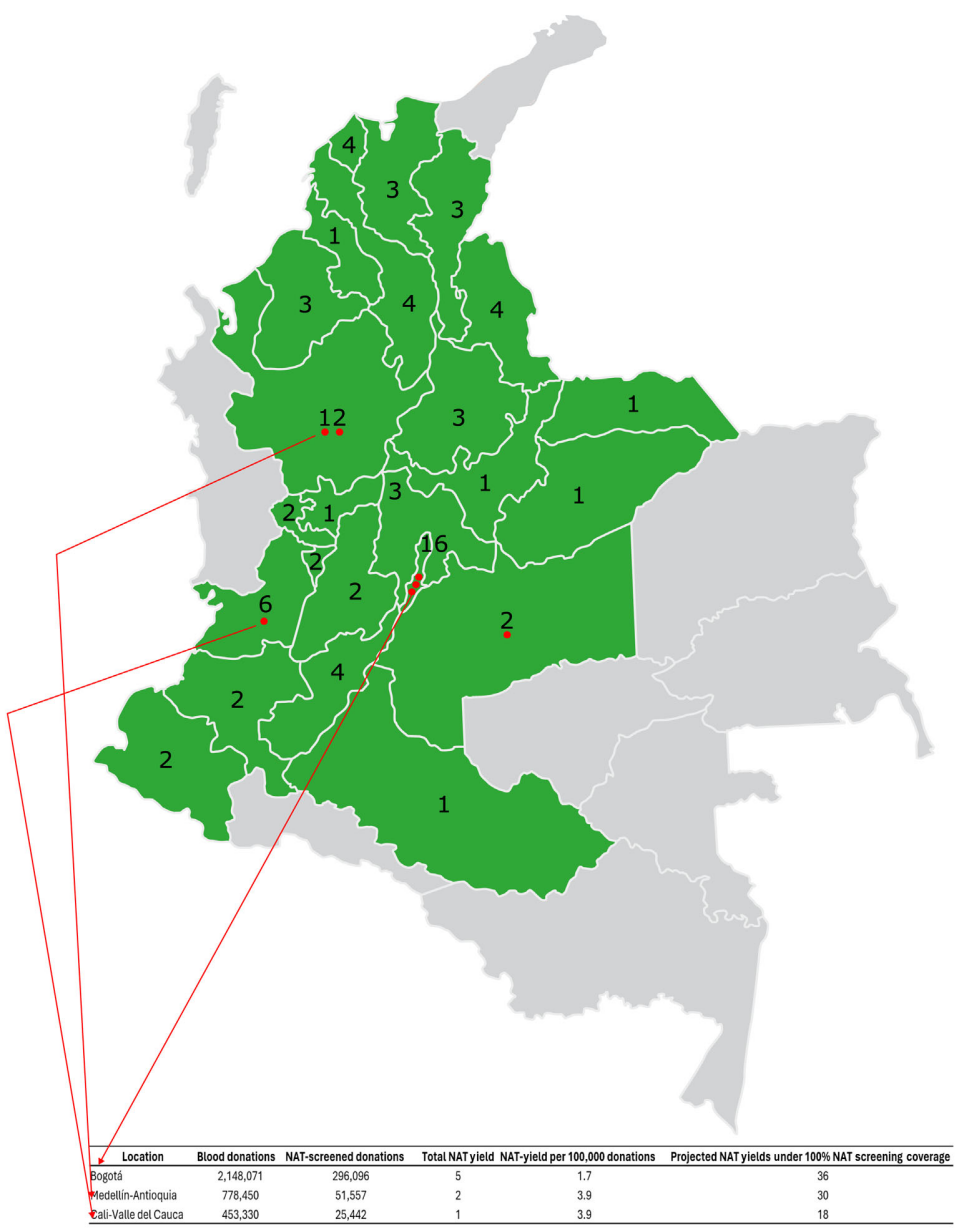
Map of Colombia showing the distribution of nucleic acid testing (NAT) platforms. The numbers indicate the number of blood banks in each region. Grey areas represent regions without blood banks, while green areas highlight departments with the highest blood collection. Red dots mark the locations of blood banks that have implemented NAT. Red arrows indicate the names of the regions where NAT‐yield cases were identified. A complementary table is included to estimate the total number of NAT‐yield cases that might have been detected if NAT screening had been applied to all donations collected in those three regions.

Between 2018 and 2024, a total of 6,435,189 blood units were collected, of which 444,937 units (6.9% of the total) were screened using NAT across 11 of the 84 blood banks (Figure 2, Tables 1 and 2). During this period, CLIA/ECLIA/ELISA screening failed to detect 10 NAT‐yield cases: four for HIV, three for HBV and three for HCV, with 80% of these cases occurring in male donors (Table 3). This indicates an estimated rate of 2.3 NAT‐yield per 100,000 donations. The adoption of NAT increased over time, from 4.3% of blood units tested in 2018 to 12.8% by the end of 2024.

**TABLE 1:** Annual national blood collection and nucleic acid testing screening coverage by year.

Year	National blood collection (*N*)	Units screened by NAT (*N*)	Percentage of national collection (%)
2018	857,736	36,595	4.3
2019	916,444	40,949	4.5
2020	796,893	38,646	4.8
2021	900,651	46,955	5.2
2022	968,547	57,111	5.9
2023	999,585	97,433	9.7
2024	995,333	127,248	12.8
Total	6,435,189	444,937	6.9

Abbreviation: NAT, nucleic acid testing.

**TABLE 2:** Nucleic acid testing screening implementation and nucleic acid testing‐yield results by blood bank and year.

Year	Blood bank	Collected units	Units screened by NAT (*N*)	Percentage of Bank's donations screened by NAT (%)	HIV	HBV	HCV
					NY	NY	NY
2018	1	33,924	33,924	100.0	1	1	0
	2	56,101	2671	4.8^a^	0	0	0
2019	1	38,831	38,831	100.0	0	0	0
	2	51,859	2118	4.1	0	0	0
2020	1	36,833	36,833	100.0	0	0	0
	2	50,562	1813	3.6	0	0	0
2021	1	42,486	42,486	100.0	0	0	0
	2	47,092	778	1.7	0	0	0
	3	54,234	3691	6.8	0	0	0
2022	1	39,145	39,145	100.0	0	0	1
	2	52,669	41	0.1	0	0	0
	3	69,701	3655	5.2	0	0	0
	4	11,517	3277	28.5	0	0	0
	5	9569	1095	11.4	0	0	0
	6	14,648	9560	65.3	0	1	0
	7	696	338	48.6	0	0	0
2023	1	38,511	38,511	100.0	0	0	0
	2	52,939	372	0.7	1	0	0
	3	71,969	3649	5.1	1	0	0
	4	12,216	12,216	100.0	0	0	1
	5	10,968	10,968	100.0	0	0	0
	6	17,799	17,799	100.0	0	0	0
	7	3984	3214	80.7	0	0	0
	8	16,082	10,704	66.6	0	0	1
2024	1	44,942	44,942	100.0	0	0	0
	3	68,644	2636	3.8	0	0	0
	4	12,463	12,297	98.7	0	0	0
	5	11,933	11,704	98.1	1	0	0
	6	21,091	21,091	100.0	0	1	0
	7	5532	5531	100.0	0	0	0
	8	15,571	14,738	94.7	0	0	0
	9	20,727	14,181	68.4	0	0	0
	10	23,588	123	0.5	0	0	0
	11	18,428	5	0.0	0	0	0
Cumulative		1,077,254	444,937	41.3	4	3	3

Abbreviations: HBV, hepatitis B virus; HCV, hepatitis C virus; HIV, human immunodeficiency virus; NAT, nucleic acid testing; NY, NAT‐yield.

^a^ Blood banks that present numbers below 100% mean that they only test a fraction of the donations. The criteria to select which units are to be tested depend on the demand of the client who requested those units for a patient.

**TABLE 3:** Demographic characteristics of individuals with a positive result by nucleic acid testing.

Virus	Age	Sex	Type of donor	Donations (*N*)	Type of donation	Location
HIV	22	Female	Repeat donor	2	Whole blood	Bogotá
	33	Male	First‐time	1	Whole blood	Medellín
	22	Male	First‐time	1	Whole blood	Meta
	48	Male	Repeat donor	7	Whole blood	Bogotá
HBV	45	Male	First‐time	1	Whole blood	Bogotá
	25	Male	First‐time	1	Whole blood	Bogotá
	34	Male	Repeat donor	5	Whole blood	Bogotá
HCV	26	Male	Repeat donor	3	Whole blood	Bogotá
	27	Female	First‐time	1	Whole blood	Cali
	39	Male	Repeat donor	2	Whole blood	Medellín

Abbreviations: HBV, hepatitis B virus; HCV, hepatitis C virus; HIV, human immunodeficiency virus.

Based on Table 2, we estimated that, between 2018 and 2024, the cumulative NAT‐yield frequency varied across blood banks. Bank 1 identified one NAT‐only reactive donation for every 91,557 donations; Bank 2, one for every 7793; Bank 3, one per 13,631; Bank 4, one per 27,790; Bank 5, one per 23,767; Bank 6, one per 24,225; and Bank 8, one per 25,442 donations. No NAT‐yield cases were detected in Banks 7, 9, 10 and 11, all of which screened fewer than 20,000 donations during the study period.

Among 342,387 donations from first‐time donors, five NAT‐only reactive cases were identified (1.46 per 100,000), compared to five NAT‐only cases among 102,550 repeat donor donations (4.88 per 100,000). The NAT‐yield was significantly lower in first‐time donors than in repeat donors (*χ* ^2^ = 4.10, *p* = 0.043). The RR was 0.30. Logistic regression confirmed this finding, with an odds ratio of 3.34 (95% CI: 0.97–11.53) for repeat versus first‐time donors. Although the CI included 1, the trend suggests a higher likelihood of NAT‐only reactivity among repeat donors. Fisher's exact test produced a marginal *p*‐value (*p* = 0.057), reinforcing the trend observed in the chi‐square analysis.

Although blood was collected in 24 regions across the country, NAT platforms were located in only 4 of them. All 10 NAT‐yield cases were identified in Bogotá, Medellín‐Antioquia, Cali‐Valle del Cauca and Meta (Table 3 and Figure 2). Considering that Bogotá, Medellín‐Antioquia and Cali‐Valle del Cauca represent the country's highest volume blood collection areas, we estimated that the NAT‐yield rate per 100,000 donations in Medellín‐Antioquia and Cali‐Valle del Cauca was 2.3 times higher than in Bogotá, despite Bogotá collecting 2.8 and 4.7 times more donations than those regions, respectively.

The NAT‐based extrapolation model estimated that approximately 135 donations within the unscreened pool may have contained HIV, HBV or HCV infections undetected by CLIA/ECLIA or ELISA, based on the observed NAT‐yield rate of 2.3 per 100,000 applied to the 5,990,252 donations not tested with NAT. Assuming an average of 2.29 components derived per whole‐blood donation, these undetected donations could have resulted in approximately 309 blood components potentially carrying infectious agents. Given that 9,637,873 blood components were transfused into 2,597,796 patients during the same period (mean: 3.7 components per patient), we estimated that up to 83 patients may have been exposed to TTIs under a serology‐only screening strategy. This corresponds to a theoretical residual risk of 8.6 infections per million components transfused (1 per 116,119 units).

In contrast, surveillance data from the High‐Cost Account reported 42 confirmed cases of HIV and 127 cases of HCV attributed to transfusion between 2019 and 2023, totaling 169 infections. Using the total number of components transfused as the denominator, the observed residual risk was 17.54 per million components (1 per 57,015 units). This value is nearly twice the risk estimated by the NAT‐based model and highlights the limitations of serological screening alone. Furthermore, only 10 of these 169 cases were recorded in the national haemovigilance system (SIHEVI‐INS), suggesting underreporting or lack of integration between clinical surveillance and transfusion safety systems.

Additionally, eight cases of occult hepatitis B were identified, defined as NAT‐HBV positive, Anti‐HBc positive and HBsAg negative. All individuals were male first‐time donors (median age: 31 years; IQR: 25.5–34.5 years) and co‐infected with HIV, while five of them also showed reactivity to syphilis.

## DISCUSSION

Between 2018 and 2024, NAT screening identified 10 cases of HIV, HBV and HCV that were not detected by standard serological assays, representing a NAT‐yield of 2.3 per 100,000 donations. Although only 444,937 donations were screened with NAT, extrapolation suggests that up to 135 additional infectious donations could have gone undetected nationwide, potentially exposing an estimated 83 patients to TTIs. The NAT‐yield varied substantially by blood bank and region, with higher rates observed in Medellín‐Antioquia and Cali‐Valle del Cauca despite lower collection volumes compared to Bogotá. Repeat donors were found to have a significantly higher NAT‐yield than first‐time donors. A comparative analysis with the national surveillance data revealed a higher observed residual risk—nearly double that estimated by modelling—and exposed major gaps in haemovigilance reporting, with only 10 of 169 transfusion‐attributed infections [12, 16, 25, 26, 27, 28, 29] captured by SIHEVI‐INS. These findings underscore the critical importance of expanding NAT coverage and improving integration between clinical and transfusion surveillance systems to enhance patient safety.

Regional and institutional variability profoundly shapes both the uptake of NAT and its yield. Although blood was collected in 24 administrative regions [1], NAT platforms were installed in just four—Bogotá, Medellín‐Antioquia, Cali‐Valle del Cauca and Meta. Notably, 90% of NAT‐yield cases came from Bogotá, Medellín‐Antioquia and Cali‐Valle del Cauca, the three regions with the highest population [30] and blood collection volumes [1]. Yet, when normalized by donations collected, Medellín‐Antioquia and Cali‐Valle del Cauca showed NAT‐yield rates 2.3 times higher than Bogotá, despite Bogotá's 2.8‐ and 4.7‐fold larger collection volumes relative to those cities. This suggests that regional epidemiological factors—such as local HIV or HCV prevalence—may amplify the window‐period burden in certain locales.

Donor characteristics further influence residual risk. In our sub‐analysis of donor types, repeat donors had 3.3‐fold higher odds of NAT‐only reactivity compared to first‐time donors. This result indicates that repeat donors—perhaps due to cumulative risk exposures or behavioural factors [22]—may contribute disproportionately to window‐period transmissions. These findings challenge the conventional assumption that first‐time donors are the primary reservoir for undetected infections [7, 31] and underscore the need for nuanced donor‐selection algorithms.

The implementation of universal NAT screening in Colombia faces several structural and economic challenges. Chief among these are the high costs associated with NAT reagents, equipment and personnel training—burdens that disproportionately affect low‐volume blood banks, approximately one‐third of which collect fewer than 5000 units annually [1], a threshold considered inefficient by the Pan American Health Organization [32]. Logistical barriers, such as the need to transport samples to centralized laboratories and ensure timely reporting of results, further hinder nationwide adoption.

Nevertheless, a formal cost–utility analysis conducted in 2018 by Vera and colleagues assessed the impact of incorporating NAT for HIV, HBV and HCV into Colombia's existing serological screening protocol (CLIA/ECLIA and ELISA) [11]. Their model projected that adding NAT would prevent approximately 64 HCV infections, 7 HBV infections and 5 HIV infections per 100,000 donations screened. The additional cost per NAT‐screened unit was estimated at USD 15 (adjusted to 2024 inflation). If NAT had been applied to all blood donations in 2018, the total additional cost would have reached approximately USD 12.2 million. However, the projected savings to the healthcare system, based on averted treatment costs, amounted to USD 29.5 million. The resulting incremental cost was negative (−USD 17.3 million), and the strategy yielded an estimated gain of 70 quality‐adjusted life years (QALYs), translating to a cost savings of USD 35,480 per QALY gained.

Further supporting the economic case, a Colombian blood bank that implemented NAT in 2024 reported a 13% increase in operational costs. Meanwhile, national HIV antiretroviral therapy coverage declined from 85.9% in 2019 to 80.4% in 2024 [12], underscoring the growing strain on HIV treatment programmes. Although HCV antiviral therapies are curative, they remain costly and have a reported failure rate of 1.5% [16].

Regionally, Ecuador has maintained near‐universal NAT since 2009 via the Red Cross [33, 34], and Panama initiated NAT in select centres in 2018 [35]. These precedents illustrate that political will and innovative financing—such as cost‐sharing consortia or public–private partnerships—can overcome resource constraints.

Our national NAT‐yield rates—9.0 per million donations for HIV and 6.7 per million for HCV—exceed the global NAT‐yields reported by Faddy et al. [7] (3.4 HIV and 1.6 HCV per million) and those in Brazil [8] (4.6 HIV and 2.1 HCV per million) or Argentina (1.0 HIV and 3.5 HCV per million) [36]. Several factors could account for these findings. First, the underlying prevalence of both HIV and HCV in the Colombian donor pool may be elevated, reflecting regional ‘hotspots’ where community transmission remains substantial [12, 16]. Second, donor‐selection practices—particularly the inclusion of repeat donors from high‐risk areas or targeted recruitment campaigns—may inadvertently enrich the screened population for window‐period infections. Third, operational differences in NAT implementation, such as smaller pool sizes or more sensitive nucleic acid platforms, can increase the likelihood of detecting low‐level viraemia that serological assays miss. Fourth, variability in assay protocols and staff training across institutions may affect detection thresholds and consistency of results. Finally, enhanced linkage between laboratory records and surveillance databases in Colombia may lead to more complete capture of NAT‐only reactive cases, whereas other settings might underreport or filter out such results. Together, these epidemiological and operational nuances plausibly drive the elevated NAT‐yields observed in our national data.

It is noteworthy that in Argentina [36], Brazil [8] and Colombia, the rates of serological window‐period donations for HBV were lower (9.6, 7.2 and 6.7 per million donations, respectively) than that reported by Faddy et al. (66.7 per million donations) [7]. Several factors could explain these differences. First, the high vaccination coverage against HBV in Argentina, Brazil, and Colombia since 2001 [37] (HBV birth‐dose and three‐dose coverage exceed 95% [38]) may contribute to lower rates of serological window‐period donations. Second is the specific genotypes of the virus circulating in the country. For example, genotype F predominates in Colombia [39], and while validated NAT assays are designed for broad genotype detection [23], theoretical concerns exist regarding primer–probe mismatches affecting pooled sample sensitivity. Although no direct evidence from Colombia confirms reduced NAT sensitivity for genotype F, this possibility warrants further investigation. Third is the real differences in HBV prevalence, especially in Africa and Asia [40, 41], as well as differences in screening practices.

Despite our extrapolation, several limitations warrant caution in interpretation. First, the NAT‐based model presumes a uniform yield rate across unscreened donations; in reality, epidemiological heterogeneity may produce localized deviations. Second, aggregate data precluded finer stratification by age, sex or socio‐economic status, which could modulate infection risk. Third, the discrepancy between the modelled residual risk (1 NAT‐yield per 116,119 units) and the observed rate based on surveillance data (1 per 57,015 units) may reflect substantial underreporting within SIHEVI‐INS, which relies primarily on passive reporting by clinicians [42]. In Colombia, there is no systematic post‐transfusion follow‐up of patients, limiting the ability to detect asymptomatic or delayed TTIs—particularly in the case of HBV and HCV. Furthermore, infections identified post transfusion may be misattributed to alternative transmission routes such as sexual contact, perinatal exposure or injection drug use, making it more difficult to establish transfusion as the likely source. These limitations in post‐transfusion surveillance likely contribute to the observed gap between modelled and reported TTI rates.

Indeed, historical haemovigilance data in Colombia have documented lower TTI rates than those reported by peer nations [43]. Since the establishment of the Colombian haemovigilance programme in 2010, no cases of transfusion‐transmitted HBV or HCV have been officially reported, despite the High‐Cost Account attributing at least 127 cases of transfusion‐transmitted HCV. In contrast, SIHEVI‐INS has documented a transfusion‐transmitted HIV rate of 1 per 963,787 blood components—approximately 1.5 times higher than the theoretical risk estimated in countries such as the United States, France, United Kingdom and Canada (1 per 1.5 million units) [44, 45], where NAT screening is mandatory. These inconsistencies underscore systemic gaps in case detection and reporting, likely exacerbated by fragmented data flows between blood centres, hospitals and public‐health registries.

Taken together, our findings support an urgent call for coordinated policy action to strengthen blood safety in Colombia. First, universal implementation of NAT screening should be prioritized, beginning with regions that have the highest NAT‐yield rates and donor populations at elevated risk, such as repeat donors. Second, improved integration of haemovigilance systems is essential; linking SIHEVI‐INS with national high‐cost disease registries would facilitate more accurate tracking of TTIs. Third, consolidation of blood banks and resource‐sharing strategies—particularly through regional consortia—could help reduce the financial burden of NAT implementation by enabling bulk procurement and centralized testing. Finally, targeted surveillance and epidemiological research, including case–control studies, are needed to refine predictive models and better understand the demographic and behavioural factors associated with window‐period infections. These measures, collectively, would significantly enhance the safety, efficiency and equity of the national blood supply.

In an era of escalating viral epidemiology [12, 13] and constrained resources, strategic deployment of NAT—coupled with data harmonization and targeted surveillance—offers the most effective means to close the window‐period gap and safeguard the blood supply. Accelerating these reforms will not only reduce the residual risk of TTIs but also reinforce public trust in transfusion safety and uphold the constitutional right to health protection in Colombia.

## CONFLICT OF INTEREST STATEMENT

The authors declare no conflicts of interest.

## Data Availability

The data that support the findings of this study are available on request from the corresponding author. The data are not publicly available due to privacy or ethical restrictions.
